# Incidental Pulmonary Nodule (IPN) Programs Working Together with Lung Cancer Screening and Artificial Intelligence to Increase Lung Cancer Detection

**DOI:** 10.3390/cancers17071143

**Published:** 2025-03-28

**Authors:** Luv Purohit, Amy Kiamos, Sundas Ali, Andres M. Alvarez-Pinzon, Luis Raez

**Affiliations:** 1 Memorial Cancer Institute, Memorial Healthcare System, Pembroke Pines, FL 33026, USA; akiamos@mhs.net (A.K.); sunali@mhs.net (S.A.); andralvarez@mhs.net (A.M.A.-P.); lraez@mhs.net (L.R.); 2 Institute for Human Health and Disease Intervention, Florida Atlantic University (FAU), Boca Raton, FL 33458, USA; 3 Pulmonary Division, Memorial Healthcare System, Hollywood, FL 33021, USA; 4 Primary Care Division, Memorial Healthcare System, Hollywood, FL 33021, USA

**Keywords:** lung cancer screening, incidental pulmonary nodule, artificial intelligence, IPN clinic, lung cancer detection

## Abstract

Current lung cancer screening guidelines in the United States fail to identify many individuals at risk of developing the disease. Additionally, existing healthcare infrastructure has been leveraged to establish IPN clinics, a promising approach to addressing the limitations of current screening guidelines. Early-stage lung cancer is frequently diagnosed because of the incidental detection of pulmonary nodules on clinically indicated chest CT scans, particularly in the absence of formal screening programs. While artificial intelligence (AI) systems for lung cancer detection have demonstrated significant advancements in medicine, their clinical validation in screening settings remains limited. This review will discuss the pivotal trials underpinning the United States Preventive Services Task Force (USPSTF) recommendations for lung cancer screening, which have shaped the current guidelines for at-risk populations. We will explore recent studies investigating the role of AI in enhancing lung cancer screening efforts, highlighting how AI has the potential to improve early detection, streamline workflows, and reduce false positives and negatives in screening processes. This review will present the lung cancer screening rates at our institution, with a specific focus on the validation and integration of AI-driven technologies into our established screening programs. Using AI algorithms, we have validated enhanced detection capabilities through retrospective analysis of historical patient data, demonstrating significant improvements in identifying high-risk individuals and early-stage malignancies. These AI models, validated through rigorous cross-validation methods and clinical trials, have proven to outperform traditional screening approaches in sensitivity and specificity. The integration of these AI technologies within the lung cancer screening framework not only optimizes existing programs but also expands access to screening, improving early detection rates and ultimately leading to better patient outcomes. Through continuous validation and refinement, we aim to solidify AI’s role in transforming lung cancer detection and patient care. Through ongoing validation and implementation, AI can play a crucial role in transforming lung cancer screening practices, ultimately contributing to earlier diagnosis and improved patient survival.

## Simple Summary

Despite well-established national screening guidelines, adoption of lung cancer screening in the United States has been slow. As of 2024, only 3–10% of eligible Americans are screened, leaving many at risk of developing late-stage lung cancer. We have shown that coupling artificial intelligence with integrated pulmonary nodule (IPN) programs can significantly improve lung cancer detection rates. Using this method, our program has diagnosed 7 to 10 times more patients compared to traditional screening methods. Leveraging AI in lung cancer screening and IPN programs can significantly enhance early detection, survival rates, and research outcomes.

## 1. Introduction

In 2024, an estimated 234,580 new cases of lung cancer were diagnosed in the United States, making it the third most common malignancy. However, with approximately 125,070 expected deaths, lung cancer will remain the leading cause of cancer-related mortality in the country [1]. With a 5-year survival of 26.7% observed between 2014 and 2020, lung cancer lags in survival statistics compared to the leading malignancies of breast and prostate cancer [1]. This can be partially explained by the late stages of lung cancer at the time of initial diagnosis. The 5-year survival rate of 63.7% indicates that curative-intent surgery or radiation therapy are frequently options for early-stage localized lung cancer. Conversely, the 5-year survival rates for regional disease and distant metastasis experience a significant decline, with the former at 35.9% and the latter at only 8.9%. This highlights the critical importance of early detection, as 47.8% of lung cancer patients were diagnosed with distant disease between 2012 and 2021 [2]. The search for lung cancer screening models has been ongoing as the incidence of lung cancer began to climb in the 1900s [3]. The landmark Mayo Lung Project, conducted between 1971 and 1984, sought to determine whether intensive cancer screening in high-risk patients could reduce lung cancer mortality [4]. Approximately 11,000 men aged 45 to 80, each smoking at least one pack of cigarettes per day, underwent an initial screening phase that included both a chest X-ray and sputum cytology. No evidence of malignancy was detected in 9211 participants, who were subsequently assigned to either a control or close-surveillance group. The control group received annual chest X-rays and sputum cytology, while the close-surveillance group underwent these screenings every four months. The close-surveillance group had 206 cancer diagnoses, with 83 being early-stage (resectable) and 112 late-stage (unresectable). The 5-year survival rate for this group was 33%, and the overall mortality was 3.2 per 1000. In the control group, 160 cancers were diagnosed, 41 early-stage and 109 late-stage, with a 5-year survival rate of 15% and an overall mortality of 3 per 1000. Although the study group showed a significant increase in 5-year survival, the primary outcome of reducing overall lung cancer-related mortality was not achieved. The authors of this study suggest that the absence of an observed lung cancer-related mortality reduction was partly due to the study being powered to detect only a minimum of a 50% reduction in mortality. Consequently, it is possible that smaller decreases in mortality were not detectable. According to other authors, the absence of an overall mortality difference may be attributed to overdiagnosis [5]. Overdiagnosis is a condition in which a malignancy is detected during a screening that is unlikely to result in symptoms or affect the patient’s health. If the malignancy is non-fatal, the survival rates are not improved by these treatments, even though increased diagnoses can result in more treatments. At the conclusion of the follow-up period, an excess of 46 cases in the study arm indicated that the radiographic or cytologic findings detected may not have resulted in any clinically relevant disease. This suggests that the excess participants may have experienced excessive anxiety and invasive interventions. Additional research has been conducted to evaluate alternative screening modalities in response to these finds and the increasing prevalence of lung cancer.

The National Lung Screening Trial (NLST) was a pivotal randomized controlled trial conducted from 2001 to 2004. Its main objective was to evaluate whether low-dose computed tomography (LDCT) scans of the chest could reduce lung cancer mortality compared to chest X-rays [6]. The study involved 53,454 participants aged 55 to 74, all with a smoking history of at least 30 pack-years, either current smokers or those who had quit within the past 15 years. Participants were randomly assigned to either receive annual chest X-rays or annual LDCT scans over a period of three years. The rate of positive screening tests was 24.2% with LDCT and 6.9% with radiography over the three years. The incidence of lung cancer was 645 cases per 100,000 person-years (totaling 1060 cases) in the LDCT group, compared to 572 cases per 100,000 person-years (totaling 941 cases) in the chest X-ray group. Lung cancer mortality was 247 deaths per 100,000 person-years in the LDCT group and 309 deaths per 100,000 person-years in the chest X-ray group, indicating a statistically significant 20.0% relative reduction in mortality with LDCT screening compared to chest X-ray. In contrast to the Mayo Lung Project study, NLST revealed that overall lung cancer mortality was significantly reduced by 6.7% in the LDCT group compared to the chest X-ray group [6]. Extended follow-up of the NLST revealed preserved lung cancer mortality benefit in the LDCT group [7]. A modest but statistically significant increase in lung cancer incidence in the LDCT arm suggesting overdiagnosis was observed in this follow-up; however, it did not negate the preserved mortality benefit. In 2013, the 2013 USPSTF guidelines recommended an annual LDCT scan for individuals aged 55 to 80 who have a 30-pack-year smoking history and currently smoke or have quit within the past 15 years because of the impact of this trial and others. The adoption of lung cancer screening has been slow, despite the recommendation [8]. A substantial number of high-risk individuals were not included in the USPTF eligibility criteria due to their limitations [8]. Additionally, socio-economic and racial disparities regarding screening are the result of significant disparities in access to screening infrastructure across the United States. The European counterpart to the NSLT trial was the NELSON lung cancer screening study, a major European randomized controlled trial, which evaluated whether LDCT screening could reduce lung cancer mortality in high-risk individuals [9]. The study was conducted in the Netherlands and Belgium and enrolled 15,792 participants, primarily men aged 50–75, with a smoking history. The participants were randomized into screening and control groups, with the screening group receiving LDCT at intervals in years 1, 2, 4, and 6. The study showed a 24% reduction in lung cancer mortality among men and a significant 33% reduction among women after 10 years of follow-up compared to the control group. The trial uniquely employed a volumetric nodule management system that classified nodules by size and volume-doubling time (VDT). This approach allowed for more accurate identification of malignant nodules while reducing false positives. For instance, only 2.6% of participants had a positive baseline result, with a false-positive rate of 64.3%, considerably lower than other studies like the NLST. Additionally, the detection rate for lung cancer in the NELSON study was 0.9% at baseline, with a sensitivity of 94.6% and a negative predictive value of 99.7%. The NELSON trial’s results, in general, confirmed the efficacy of LDCT screening in reducing lung cancer mortality by identifying cancer at earlier, more treatable stages, particularly when combined with advanced nodule management protocols. The guidelines for lung cancer screening in Europe were significantly impacted by these recent findings.

As previously mentioned, the primary focus of screening has been the smoking history of patients. However, in the United States, approximately 10% to 20% of lung cancers or 20,000 to 40,000 lung cancers annually occur in individuals who have never smoked or have smoked fewer than 100 cigarettes in their lifetime. This means that a substantial number of individuals are at risk of developing late-stage, incurable lung cancer, as they are not accounted for in the current screening guidelines.

The TALENT study, conducted at 17 tertiary centers in Taiwan, used LDCT screening among never-smokers with or without a family history of lung cancer to assess the efficacy of this approach [10]. The study enrolled 12,011 participants between December 2015 and July 2019, with 93.3% being never-smokers and 50% having a family history of lung cancer. Eligible participants underwent baseline LDCT, followed by annual and biennial screening for up to 8 years. LDCT scans were positive in 17.4% (n = 2094) of participants, with lung cancer diagnosed in 318 individuals (2.6%). Of those, 257 (2.1%) had invasive lung cancer, and 61 (0.5%) were diagnosed with adenocarcinoma in situ. This study has a high detection rate for invasive lung cancer at a higher rate (2.1%) than U.S. (NLST: 1.1%) and European (NELSON: 0.9%) studies in smokers. The majority (77.4%) of participants diagnosed with lung cancer were in stage I, highlighting the effectiveness of early detection. It was seen that the prevalence of Lung cancer was higher among participants with a family history (2.7%) compared to those without (1.6%) of lung cancer. The risk of lung cancer increased with age, particularly in invasive forms, while the detection of adenocarcinoma in situ remained stable. The analysis determined that individuals who had a first-degree relative (particularly a mother or sibling) were at an increased risk of developing lung cancer. In the multivariable analysis, the risk of lung cancer was significantly elevated by female sex, a family history of lung cancer, and an age over 60. Interestingly, passive smoke exposure, cumulative exposure to cooking, and cooking without ventilation were not significant risk factors after adjustment. In the detection of lung cancer, LDCT screening exhibited a high specificity (84.6%) and sensitivity (92.1%), with a positive predictive value (PPV) of 14.0% and a negative predictive value (NPV) of 99.7%. Given the slower progression rates of these lesions, the high proportion of adenocarcinoma in situ (AIS) diagnoses (19.2%) underscores concerns regarding overdiagnosis. Additionally, 17.9% of lung cancer participants reported multiple primary tumors, with the highest risk being associated with a family history of the disease and a lower body mass index (BMI). These results underscore the pressing necessity for additional research to customize screening programs for high-risk populations. To guarantee that screening initiatives are both effective and efficient, it is imperative to refine risk prediction models and address overdiagnosis, thereby minimizing unnecessary interventions and maximizing their early detection. 

Beyond traditional LDCT screening, leveraging pulmonary nodule detection presents a promising complementary approach. This method utilizes existing healthcare infrastructure to identify patients with potentially malignant lesions, including those who may not qualify for or have access to LDCT screening. To improve lung cancer outcomes, the American Cancer Society National Lung Cancer Roundtable (ACS NLCRT) convened a multistakeholder group to identify and address gaps in lung nodule management. This initiative focuses on key aspects, including risk stratification, diagnostic evaluation, care implementation, national quality improvement efforts, and health equity. By addressing these gaps, the goal is to create more inclusive and effective care models that enhance outcomes for patients at risk of or diagnosed with lung cancer. 

## 2. Methodology

To comprehensively assess the current landscape and future directions of lung cancer screening, we conducted a structured search of the literature across multiple databases, including PubMed, MEDLINE, and Scopus. Our search strategy targeted studies covering key aspects of lung cancer screening, such as screening modalities, eligibility criteria, implementation strategies, and emerging technologies. We limited the search to peer-reviewed articles published from January 2013 through February 2025. We placed special emphasis on studies appearing after 2021, when the U.S. Preventive Services Task Force (USPSTF) updated its lung cancer screening guidelines. We did include pivotal trials to narrate the history of lung cancer screening programs. For the database queries, we used various combinations of keywords related to lung cancer screening. These included terms such as “lung cancer screening”, “low-dose CT (LDCT)”, “screening guidelines”, “early detection”, “risk stratification”, “artificial intelligence”, “biomarkers”, and “implementation barriers”. In addition to the database search, we reviewed relevant policy statements, clinical guidelines, and real-world data from lung cancer screening programs in the United States, Europe, and Asia.

Inclusion criteria encompassed a range of publication types, including original research studies, systematic reviews, meta-analyses, clinical trials, guideline statements, and notable expert commentaries. We included sources only if they focused on lung cancer screening in adults aged 50 years or older with a substantial smoking history. Conversely, we excluded any articles not published in English, as well as case reports, conference abstracts, or studies not directly relevant to lung cancer screening.

Multiple authors reviewed each article to assess its quality, relevance, and recency. We then synthesized the findings into the following five key thematic domains that recurred across the selected literature: historical evolution of lung cancer screening guidelines; current screening practices and outcomes; health disparities and access to screening; advances in risk prediction models and non-imaging biomarkers; and future directions in screening, including AI-based imaging tools, blood-based screening assays, and personalized screening strategies. By structuring our review around these key themes, we aim to provide a balanced synthesis of the current evidence and highlight knowledge gaps that could inform future lung cancer screening and early detection efforts.

## 3. Incidental Pulmonary Nodule Programs

The frequency of CT utilization is rising rapidly, with 28 studies ordered for every 100 patients seen in the emergency department, an increase from 18 per 100 in 2006 [11]. IPN programs have leveraged this rise in CT utilization to detect pulmonary nodules on all CT scans, regardless of the indication for ordering the scan. In other words, IPN programs do not wait for patients to get screened, instead they take advantage of imaging being obtained for various reasons to capture a wider population of patients.

The Detecting Early Lung Cancer in the Mississippi Delta Cohort (DELUGE) trial studied the effects of guideline-based management of incidentally detected pulmonary nodules [12]. One of its primary objectives was to evaluate the disparities in lung cancer diagnosis stage, surgical resection rates, and 3- and 5-year survival outcomes among various early detection methods. In addition, the investigation assessed whether patients diagnosed using these methodologies would have been eligible for an LDCT scan in accordance with the 2021 USPSTF guidelines. The participants were divided into the following three groups: those in the LDCT group who met the 2021 USPSTF criteria, the incidental nodule (LNP) group who were contacted based on radiology reports of suspicious lung nodules that were performed for non-cancer-related reasons, and patients who were discussed by the Baptist Memorial Health Care Corporation (BHMCC) multidisciplinary thoracic oncology program’s tumor board, which met weekly to review suspected lung cancer cases. In the LDCT group, 156 cases of lung cancer were diagnosed, 772 cases in the LNP group, and 1150 cases in the BHMCC group. A review of the primary outcomes is described in Table 1.

The DELUGE trial made several key observations. It showed a 4:5:1 ratio of patients diagnosed with Stage I or II disease between the LDCT, LNP, and BHMCC groups, solidifying the notion that early detection programs are effective. This translated to improved survival among the LDCT and LNP groups compared to the BHMCC group. Additionally, there was a 5:1 enrollment rate between the LNP group and LDCT group, with the LNP group including more participants of minority demographics. Coupled with the observation that approximately 50% of patients diagnosed with lung cancer in this study would not have been eligible for LDCT per the 2021 USPSTF criteria, these data suggest that integrating guideline-based management of IPNs can capture a significant portion of at-risk individuals.

As mentioned earlier, the frequency of CT utilization is rising rapidly. The large amount of real-time data excites investigators because incidental findings, though not urgent, may later help answer questions as a patient’s care evolves over time. However, manually processing these data is challenging and cumbersome. With the advent of AI, the processing of massive amounts of real-time data has come within reach. The implementation of AI alongside an IPN management program can potentially improve cancer detection rates. 

## 4. Implementing Artificial Intelligence

Conventional lung cancer screening protocols frequently omit individuals who do not belong to high-risk categories, including nonsmokers or those with genetic predispositions. The utilization of AI in the detection of IPNs enhances screening accessibility, uncovering cases that conventional methods may overlook. Utilizing routine imaging for IPN detection, AI enhances healthcare resource distribution and alleviates the financial strain on resource-limited health systems, thereby improving the feasibility and sustainability of cancer screening initiatives. Radiologists often encounter IPNs, and predicting the likelihood of malignancy can be challenging due to interpretative variability and the need for monitoring small nodules. In a study by Torres et al., the radiologist did not detect up to 35% of small nodules after a single examination [13]. The International Early Lung Cancer Action Program conducted a study analyzing the screening CT scans of confirmed lung cancer patients. The study’s results revealed that 75% of the verified cancerous nodules were retrospectively identifiable in the previous CT scan [14]. 

John McCarthy, a computer scientist, was the first to introduce Artificial Intelligence (AI) in 1956. McCarthy defined it as “the science and engineering of creating intelligent machines” [15]. AI has been increasingly integrated into a variety of industries, including healthcare, because of its capacity to replicate human intelligence and critical thinking [16]. The development of computer-aided diagnostic (CAD) systems has had a significant application in healthcare. These systems aid radiologists in the detection and classification of pulmonary nodules. By reducing inter-reader variability, these systems enhance the accuracy of radiological lesion assessment. AI has been utilized in pulmonary nodule detection since the 1980s, with early CAD systems paving the way for more sophisticated technologies [17]. 

Deep learning (DL) has emerged as a powerful tool that employs sophisticated mathematical algorithms to process and synthesize data due to advancements in AI. Deep learning is increasingly being explored for its ability to accurately classify pulmonary nodules based on malignancy prediction, especially regarding malignancy risk stratification. AI technology improves the accuracy and precision of image interpretation by effectively analyzing extensive amounts of imaging data and identifying nuanced details frequently overlooked by conventional diagnostic techniques. Its capacity for precise quantification renders it an indispensable resource for radiologists. Moreover, AI enhances precise risk assessment for malignancies, accelerates confirmatory diagnoses, reduces false negatives, and mitigates psychological distress for patients with benign pulmonary nodules. This capability is particularly impactful in rural and low-income regions, enhancing diagnostic precision in primary care [17,18,19,20]. 

DL-based systems provide a streamlined and efficient diagnostic process by directly analyzing imaging and patient information, in contrast to traditional systems that necessitate manual data input. For instance, the lung cancer prediction convolutional neural network (LCP-CNN) generates individualized malignancy risk scores for each nodule that is evaluated. In addition to enhancing the classification of pulmonary nodules, this capacity to rapidly analyze data, identify patterns, and develop systematic algorithms has resulted in groundbreaking discoveries, including the identification of DNA variants that predict disease risk [18].

Radiomics is a rapidly developing field of radiologic imaging that employs contemporary AI to acquire quantitative imaging features that predict disease diagnosis and prognosis, thereby assisting clinical decision-making [18]. Radiomics employs advanced techniques to extract data from imaging, such as the shape, size, and texture characteristics that can be used to predict the likelihood of lung nodule malignancy [19]. Radiomics enhances the detection of malignant pulmonary nodules by identifying subtle nodule characteristics that are invisible to the human eye and synthesizing complex Hounsfield unit distributions for textural analysis [18]. Ardila et al. conducted a study in which they compared the detection of lung cancer on LDCT with a DL algorithm and traditional radiologists. The results showed an absolute reduction of 11% in false positives and 5% in false negatives when compared to the six diagnostic radiologists [20]. Moreover, AI can monitor the changes in IPN over time, enabling the continuous monitoring of growth progression. The rapid and accurate diagnosis of pulmonary nodules and the prediction of lung cancer risk are achieved through the statistical analysis of these radiomic features [21].

As AI advances, the field of radiomics grows, allowing us to perform increasingly sophisticated data analyses. Radiomics can predict the immune phenotype of pulmonary tumors based on radiomic signatures and assess for predictors of immunotherapy treatment efficacy [22]. Tumor characterization analysis with radiomics has also been shown to predict somatic mutations. For example, Wang et al. revealed an association between tumor spiculation and KRAS mutation [23]. The incorporation of AI into medicine is exciting, with extensive ongoing research. Further studies are needed with radiomics to help tailor our treatment of lung cancer and hopefully improve future outcomes using AI. 

## 5. Incidental Pulmonary Nodule Programs with Artificial Intelligence

IPN programs have capitalized on the rise in CT utilization to enroll a patient population that is not currently accessible under the current lung cancer screening guidelines, as previously mentioned. Further, AI and DL have advanced by assisting radiologists in the detection of lung cancer. Numerous institutions have recognized the untapped potential of integrating AI into IPN programs to optimize the detection of lung cancer and decrease referral times. The University of Colorado was among the first to implement AI-driven IPN programs. Within the first seven months of its implementation, they reported a remarkable yield of 263 cancer diagnoses [24]. Similarly, Geisinger Health has created the System to Track Abnormalities of Importance Reliably (STAIR) program. This program refers incidental nodules that are diagnosed using AI to the STAIR program, where they are subsequently monitored in accordance with established guidelines. As a result of the implementation of this program, the average referral time from the time of nodule identification to the evaluation by a physician was reduced from three months to nine days [25]. At our institution, the integration of an AI-based screening tool at the Memorial Cancer Institute in South Florida resulted in a tenfold increase in lung cancer detection rates. This substantial improvement can be attributed to the AI-driven model’s ability to accurately identify incidental pulmonary nodules (IPNs) through a seamless integration with our Electronic Medical Records (EMRs) system. Between February 2023 and July 2024, we analyzed discovery reports from CT scans, identifying 4186 cases of incidental IPNs, all of which were monitored using our AI-powered IPN screening tool.

The AI model was validated using a combination of retrospective analysis and real-time clinical data, employing cross-validation techniques to assess performance in terms of sensitivity, specificity, and positive predictive value (PPV). During the validation process, the AI model demonstrated a significant improvement over traditional radiological assessment in detecting early-stage lung cancer, with a sensitivity rate of 92% and a specificity of 87%. The tool’s ability to identify high-risk patients with a high degree of accuracy was pivotal in refining patient follow-up protocols, ensuring that at-risk individuals were closely monitored. As a result, 96 patients were diagnosed with lung cancer following AI-guided identification and tracking of their IPNs. This model, through its continuous learning from clinical feedback, has proven to enhance early detection, optimize clinical workflows, and ultimately improve patient outcomes.

**Figure 1:**
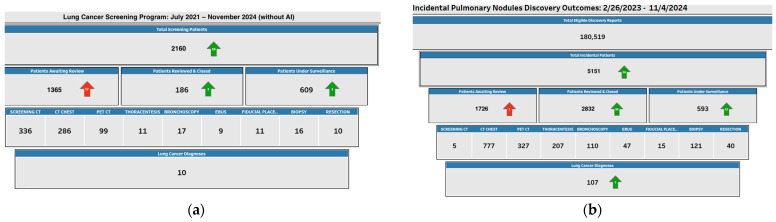
Memorial Cancer Institute Incidental Pulmonary Nodule Program. (**a**) On the left, the figure depicts the data from our traditional lung cancer screening program between July 2021 and July 2024 prior to the use of our combined IPN and AI model. (**b**) On the right, the figure depicts the data from our combined IPN and AI model between 26 February 2023 and 4 November 2024. There is a marked increase in lung cancer detection in the IPN and AI model from 10 to 107 diagnoses after only approximately 20 months of implementation.

As seen in Figure 1, this is a tenfold increase in comparison to the results that were observed prior to the implementation of the AI tool. Prior to its implementation, only ten cases of lung cancer were identified among 1925 patients who underwent screening and follow-up in our traditional lung cancer screening program. AI technology has the potential to significantly improve clinical outcomes, particularly in the early detection of potentially life-threatening conditions like lung cancer, as evidenced by the stark contrast in detection rates. The significant increase in detection is an indicator to the effectiveness of AI in enhancing the efficiency, consistency, and accuracy of screening processes. AI may assist radiologists identify incidental pulmonary nodules more accurately and earlier than traditional methods by automating the analysis of vast amounts of imaging data. The findings from our study emphasize the transformative impact of AI tools in enhancing lung cancer screening programs, particularly within a community healthcare system and cancer center of excellence. The AI-powered module deployed at the Memorial Cancer Institute in South Florida was specifically designed to integrate seamlessly into our established clinical workflows, optimizing lung cancer detection in a community-based setting where resource constraints and varied levels of clinical expertise exist.

The AI model utilized in our center incorporates advanced machine learning algorithms trained on extensive datasets, including thousands of CT scans from both community and specialized healthcare settings. It was validated through a rigorous process, using a cohort of patients with known clinical outcomes to evaluate its accuracy in detecting incidental pulmonary nodules (IPNs). Through retrospective cross-validation and real-time implementation, the AI tool demonstrated significant improvements in both sensitivity and specificity, with a marked reduction in false-negative rates, which are critical for early-stage lung cancer diagnosis. The module assesses key radiological features, such as nodule size, morphology, margins, and attenuation values, parameters that have been correlated with malignancy risk. These features are analyzed using deep learning techniques, which allow the system to detect subtle patterns and variations in nodules that may not be apparent to less experienced clinicians or in busy clinical environments typical of community healthcare systems. This provides a standardized, evidence-based approach that minimizes inter-reader variability, especially important in a community cancer center where radiologists and pulmonologists may have different levels of expertise.

The system’s ability to assist less experienced clinicians in accurately identifying and stratifying patients based on risk has been a pivotal aspect of its success. It supports clinicians by providing objective, reproducible assessments of nodules, allowing for better-informed decisions regarding follow-up protocols, biopsy, or further imaging. In our study, the AI tool identified 4186 IPN cases, with 96 confirmed diagnoses of lung cancer, demonstrating its efficacy in early-stage detection and risk stratification. By streamlining the diagnostic process and reducing biases associated with human interpretation, the AI module has enhanced the overall efficiency of lung cancer screening in our cancer center of excellence. Moreover, its integration into a community healthcare system has expanded access to high-quality diagnostic tools, ensuring that underserved populations, often at greater risk of late-stage lung cancer, receive timely and accurate diagnoses.

In summary, the continued incorporation of AI technologies within community-based cancer centers is essential to improving patient outcomes, reducing health disparities, and driving precision oncology forward. The success of this module underscores the importance of investing in AI-driven healthcare solutions that enhance both diagnostic accuracy and access to life-saving treatments for patients, particularly in underserved or resource-limited settings.

## 6. Discussion

Lung cancer detection and treatment (LCDT) programs have faced persistent challenges since their inception, with progress impeded by low screening uptake and modest success rates. To improve pulmonary nodule management, it is imperative to emphasize high-quality research, including randomized controlled trials (RCTs) and observational studies. Landmark studies such as DELUGE and TALENT underscore the urgent need to refine surveillance protocols and evaluate alternative management strategies [21,22,23]. Transitioning from static guidelines to adaptive, “living” guidelines would enable the timely incorporation of advancements like volumetric nodule assessment techniques validated through clinical research [24,25,26,27]. Such efforts would enhance patient outcomes and optimize care pathways. Furthermore, ensuring the inclusion of diverse populations in these studies is crucial for broad clinical applicability, mitigating overdiagnosis and improving risk stratification models [28,29].

While LDCT screening holds promise for lung cancer detection, significant barriers remain. Screening rates are low, and current eligibility criteria exclude many at-risk individuals, including approximately 30,000 never-smokers diagnosed with lung cancer annually. Disparities in screening access—shaped by race, ethnicity, geography, and other social determinants of health—further exacerbate inequities in care. IPN programs serve as a critical complementary strategy, identifying patients with pulmonary nodules through incidental findings on imaging studies. These programs extend screening to individuals who do not meet LDCT criteria, including those without significant tobacco exposure or those outside the defined age range [16,17,18,19].

To optimize the effectiveness of integrated pulmonary nodule (IPN) programs, addressing technological deficiencies in key areas such as nodule tracking, standardized reporting, and risk assessment is critical. The integration of AI-driven tools has shown considerable promise in enhancing the identification, classification, and management of pulmonary nodules, thus streamlining clinical decision-making. These tools can also facilitate the expansion of IPN programs to include imaging studies beyond standard chest CT, such as thoracic spine, cardiac, calcium scoring, and abdominal CT scans. These studies often incidentally capture lung-related findings, providing additional opportunities for early detection of pulmonary issues [29,30,31].

However, for these advancements to effectively contribute to improved outcomes, the successful implementation of AI tools must be grounded in the precision and accuracy of the underlying algorithms. This highlights the critical importance of not only integrating AI-driven solutions into clinical care but also incorporating them into clinical studies and prescreening protocols for cancer patients. These tools must undergo rigorous and ongoing refinement, coupled with continuous clinical validation, to ensure they remain clinically relevant, reliable, and capable of adapting to evolving patient populations and healthcare settings. In addition to integration into routine clinical workflows, expanding the scope of imaging analysis through AI can significantly enhance early detection of malignancies, enabling timely interventions that can dramatically improve patient prognosis. AI-driven models can help identify subtle radiological features and patterns that may otherwise go unnoticed, particularly in high-risk populations or in settings where access to specialized radiologists may be limited. As these technologies undergo further validation in diverse clinical environments, their ability to detect cancer at earlier, more treatable stages will become increasingly valuable, contributing to more effective and personalized cancer care strategies. Ultimately, continuous enhancement of these AI tools will be essential to harness their full potential and optimize the effectiveness of IPN programs in the clinical setting [24,32,33]. 

The integration of AI with standardized electronic health record (EHR) systems can improve the detection of nodules, longitudinal surveillance, and outcome monitoring. In addition to delivering guideline-concordant care, multidisciplinary nodule clinics improve diagnostic accuracy and improve patient-centered outcomes, thereby further supporting these initiatives. However, to optimize program implementation, targeted interventions are necessary to address obstacles such as resource variability, workforce limitations, and fragmented communication. Efforts to improve equity and quality metrics in lung nodule management must incorporate technology and foster collaboration between IPN programs, LDCT initiatives, and AI-enabled tools. Addressing disparities requires targeted research and interventions tailored to underserved populations. Establishing national quality metrics for structured nodule reporting and follow-up, as well as developing nodule registries and biorepositories, is essential for standardizing care, validating risk prediction models, and supporting clinical trials. Beyond routine screening, AI has also proven valuable in optimizing participant selection for clinical studies, automating the identification of appropriate and diverse patient populations to enhance research validity and efficiency. This dual application of AI in clinical care and research underscores its potential to address critical gaps in pulmonary nodule management, including risk stratification, tracking, and standardized reporting. 

Molecular testing is emerging as a valuable tool in lung cancer screening, with proteomic and miRNA-based blood assays enhancing risk assessment for pulmonary nodules. Nodify Lung^®^, a proteomic classifier, demonstrated high sensitivity (97%) and negative predictive value (98%) in the PANOPTIC trial, reducing unnecessary procedures [34]. Several miRNA-based assays show promise for early lung cancer detection, though none are FDA-approved. While these tests may complement low-dose CT (LDCT) screening by refining risk stratification and reducing false positives, they are not currently recommended by major oncology societies and remain confined to research settings. Future integration with AI-driven imaging could further improve detection accuracy, offering a more precise and personalized approach to lung cancer screening. However, large-scale studies are needed to validate their clinical utility and cost-effectiveness before widespread adoption.

The potential benefits of the investment, such as improved survival rates and reduced healthcare burdens associated with late-stage lung cancer, justify the investment, despite the ongoing concerns about the cost of implementing these programs. These strategies can also generate favorable returns through downstream revenue from diagnostic workups and treatments, which is facilitated by early detection [30,31,32,33]. The integration of AI algorithms with high sensitivity and specificity, in conjunction with clinician oversight for the evaluation of identified IPNs, establishes a critical foundation for optimizing treatment planning. To alleviate patient anxiety, reduce healthcare expenditures, and prevent unnecessary treatment-related adverse effects, incidental lung cancer (LC) findings detected by AI must be contextualized within the appropriate clinical framework. The current literature emphasizes the critical role of medical professionals in evaluating the clinical relevance of incidental findings during the initial stages of AI adoption by emphasizing the discrepancy between algorithmic accuracy and practical clinical utility. 

To enhance early lung cancer detection, reduce disparities in nodule management, and improve the consistency of care in early clinical trials and research, it is essential to implement comprehensive strategies aligned with national cancer objectives. These strategies should focus on overcoming technological, systemic, and equity-related challenges within the healthcare system. By addressing these challenges, the integration of AI and other innovative tools can improve the accuracy and efficiency of lung cancer screening, ensuring that all patients—regardless of socioeconomic background—receive timely and high-quality care. Technological advancements, such as AI-driven imaging analysis, must be combined with systemic efforts to standardize care protocols across diverse healthcare settings. This includes increasing access to cutting-edge diagnostic tools in underserved communities and ensuring that clinicians at all levels are trained to use these technologies effectively. Furthermore, ensuring equity in lung cancer care involves addressing disparities in access to screening, follow-up, and treatment, particularly for high-risk populations, including those from historically underserved or marginalized communities.

By focusing on these key areas—technological innovation, standardized care, and equity—we can create a healthcare ecosystem that not only enhances the early detection of lung cancer but also improves patient outcomes across diverse populations. Through these efforts, the field of lung cancer care will be better positioned to meet national cancer objectives and deliver more effective, equitable, and accessible care to all patients with pulmonary nodules [28,30,31,32,33].

## 7. Conclusions

The success of lung cancer screening cannot rely only on referrals from primary care doctors when they identify patients at risk. We have too many patients that do not fit in the USPSTF guidelines that develop lung cancer, and studies like TALENT show that they should be considered for screening. IPN programs give an opportunity to increase the number of screened patients; it is also a responsibility of healthcare providers because these CT scans are often already performed for reasons other than cancer. AI gives us a tremendous opportunity to help in IPN programs, where massive amounts of information and data are generated that make it difficult and expensive to digest only with human resources. 

Leveraging AI in lung cancer screening and IPN programs can significantly enhance early detection, survival rates, and research outcomes. Collaboration between multidisciplinary teams and AI-driven tools improves diagnostic accuracy and streamlines care. These advancements reduce healthcare burdens by optimizing patient management and resource use. Continued investment in AI solutions is essential to advance lung cancer care and improve patient outcomes. Ultimately, AI has the potential to revolutionize the fight against lung cancer.

## Figures and Tables

**Table 1:** Results of the Detecting Early Lung Cancer in the Mississippi Delta Cohort (DELUGE) trial illustrating the significant number of patients with lung cancer that would not have been eligible for low-dose CT scan screening per USPST captured by other early detection programs. This also illustrates the large proportion of early-stage lung cancer detected by all early detection programs.

Study Group	LDCT	LNP	BHMCC
Diagnosed/Total, (%)	156/5669 (3%)	772/15461 (5%)	1150/1766 (65%)
Stage I or II, (%)	10%	44%	47%
Surgical Resection, (%)	10%	42%	48%
3-year Survival	80%	64%	49%
5-year Survival	76%	60%	44%
Age, median (Q1–Q3)	68 (64–72)	69 (63–76)	68 (61–75)
White, (%)	84	71	69
Black, (%)	16	27	30
Active Smoker	72	46	41
Former Smoker	28	40	45
Never Smoker	0	13	13
Pack years—Former Smoker median (Q1–Q3)	55 (40.75–72.5)	41 (24.25–60)	20.25 (21.5–60)
Quit years—Former Smoker median (Q1–Q3)	8 (2.75–11.25)	16 (7–28)	11 (4–24)
Proportion Eligible for LDCT by USPSTF 2021 Criteria, No, (%)			
All participants	4720 (83.41)	2280 (14.75)	718 (40.66)
Patients with lung cancer	137 (91.33)	344 (49.28)	529 (52.38)
