# Correction: Purohit et al. Incidental Pulmonary Nodule (IPN) Programs Working Together with Lung Cancer Screening and Artificial Intelligence to Increase Lung Cancer Detection. *Cancers* 2025, *17*, 1143

**DOI:** 10.3390/cancers17203345

**Published:** 2025-10-17

**Authors:** Luv Purohit, Amy Kiamos, Sundas Ali, Andres M. Alvarez-Pinzon, Luis Raez

**Affiliations:** 1 Memorial Cancer Institute, Memorial Healthcare System, Pembroke Pines, FL 33026, USA; akiamos@mhs.net (A.K.); sunali@mhs.net (S.A.); andralvarez@mhs.net (A.M.A.-P.); lraez@mhs.net (L.R.); 2 Institute for Human Health and Disease Intervention, Florida Atlantic University (FAU), Boca Raton, FL 33458, USA; 3 Pulmonary Division, Memorial Healthcare System, Hollywood, FL 33021, USA; 4 Primary Care Division, Memorial Healthcare System, Hollywood, FL 33021, USA

There was an error in the original publication [1]. In Section 5, Paragraph 1, data from University of Colorado Health (UCHealth) was referenced, but it was incorrectly attributed to the University of Cincinnati (UC Health) in both the text and the citation. The incorrect sentence has been replaced with the following:

The University of Colorado was among the first to implement AI-driven IPN programs. Within the first seven months of its implementation, they reported a remarkable yield of 263 cancer diagnoses [24].

In addition, [24] has been updated to include the correct source:

24. University of Colorado. Catching Lung Cancer Earlier through AI-Powered Systems. UCHealth. 2024. Available online: https://www.uchealth.org/today/catching-lung-cancer-earlier-through-ai-powered-systems/ (accessed on 17 November 2024).

The authors apologize for any inconvenience caused and state that the scientific conclusions are unaffected. This correction was approved by the Academic Editor. The original publication has also been updated.
